# Cyclophilin D binds to the acidic C-terminus region of α-Synuclein and affects its aggregation characteristics

**DOI:** 10.1038/s41598-020-66200-9

**Published:** 2020-06-23

**Authors:** James Torpey, Jillian Madine, Amy Wood, Lu-Yun Lian

**Affiliations:** NMR Centre for Structural Biology and Institute of Systems, Molecular and Integrative Biology, University of Liverpool, Liverpool, L69 7ZB UK

**Keywords:** Protein aggregation, Intrinsically disordered proteins, Parkinson's disease

## Abstract

Cyclophilin D (CypD) is a peptidyl-prolyl isomerase expressed in the nucleus and transported into the mitochondria where it is best associated with the regulation of the mitochondrial permeability transition pore (MPTP). There are, however, other possible roles of CypD in the mitochondria which may or may not be linked with the MPTP. Alpha synuclein (αSyn) is shown here to interact directly with CypD via its acidic proline-rich C-terminus region and binding at the putative ligand binding pocket of CypD. The study shows that CypD binding with soluble αSyn prevents its aggregation. Furthermore, the addition of CypD to preformed αSyn fibrils leads to the disassembly of these fibrils. Enzymatically-compromised mutants of CypD show reduced abilities to dissociate αSyn aggregates, suggesting that fibril disassembly is linked to the increased rate of peptidyl-prolyl isomerisation catalysed by CypD. Protein aggregation in the mitochondria is increasingly seen as the cause of neurodegeneration. However, protein aggregation is a reversible process but disaggregation requires help from other proteins such as isomerases and chaperones. The results here demonstrate a possible mechanism by which CypD achieves this and suggest that disaggregation could be one of the many functions of this protein.

## Introduction

Cyclophilin D (CypD) is a mitochondrial peptidyl-prolyl isomerase that has been implicated to be involved in the mechanisms of many diseases, although it is best known as a regulator for the opening of the mitochondria permeability transition pore (MPTP). These conclusions were drawn from outcomes observed in *in vivo* studies involving the use of the potent inhibitor, cyclosporine A (CsA), and *ppif* gene knockdown mice^1,2^. In a recent study, genetic ablation of CypD resulted in a delayed onset of Parkinson’s Disease (PD) and extended lifespan of PD mice^3^. Co-immunoprecipitation experiments in the same study show very weak direct interactions between CypD and αSyn^3^. The connection between CypD and PD has not been clearly established, with the exception that mitochondrial dysfunction is a hallmark of PD^4^. PD is characterised by the formation of Lewy bodies, which are protein aggregates containing the protein alpha-synuclein (αSyn) and selective degeneration of dopaminergic neurons in the substantia nigra brain region^5,6^. Genome-wide analyses place mutations in SNCA, the gene encoding αSyn, as the top risk factor for those developing sporadic forms of the disease^7,8^. αSyn redistributes from the cytoplasm to the outer and inner mitochondrial membrane with increased accumulation in PD^9,10^. There is a correlation between αSyn entry into the mitochondria, reduced mitochondrial membrane potential (∆Ψ_m_) and mitochondria dysfunction, implicating the involvement of the MPTP^11,12^. These effects of αSyn can be rescued by the addition of CsA, a CypD inhibitor, although this could be through the role of CypD as a regulator of the MPTP rather than as a result of direct interaction between αSyn and CypD^13^.

Proteins currently known to dissociate amyloid fibrils do so in either an ATP-dependent or –independent manner. Examples of the former include Hsp70, whereas the latter mode can be found in Cyclophilin 40 (Cyp40)^14,15^. Cyp40, a cytosolic peptidyl prolyl isomerase which is known to act as a co-chaperone to Hsp90, was reported to dissociate tau, and αSyn, but not amyloid-β (Aβ) fibrils^15,16^. The effects of Cyp40 on the tau and αSyn fibrils were attributed to its peptidyl-prolyl isomerase activity. Hence, we hypothesise whether other cyclophilins have similar disaggregation capabilities. Given previous evidence of weak interactions between αSyn and CypD^3^, and the increasing evidence of αSyn localisation in the mitochondria^17^, we set out to determine how CypD interacts with αSyn and what effects these interactions might have.

CypD is smaller than Cyp40; it has a 40 amino-acid mitochondrial penetrating peptide at its N-terminuswhich is cleave off in the mature protein, and lacks the three tetratricopeptide repeats at the C-terminus. Unsurprisingly, however, the crystal structures of both proteins show significant structural similarity in the cyclophilin catalytic domain^18^. Using *in vitro* methods, CypD is shown here to have the ability to prevent αSyn aggregation as well as disrupt preformed αSyn fibrils. Catalytically compromised mutants were less able to dissociate the preformed fibrils. The acidic C-terminus region of αSyn is identified to be the main binding site to CypD. These *in vitro* results provide some mechanistic insights into how and why isomerases dissociate protein aggregates.

## Results

### CypD prevents αSyn aggregation and promotes disaggregation of preformed fibrils

**Figure 1:**
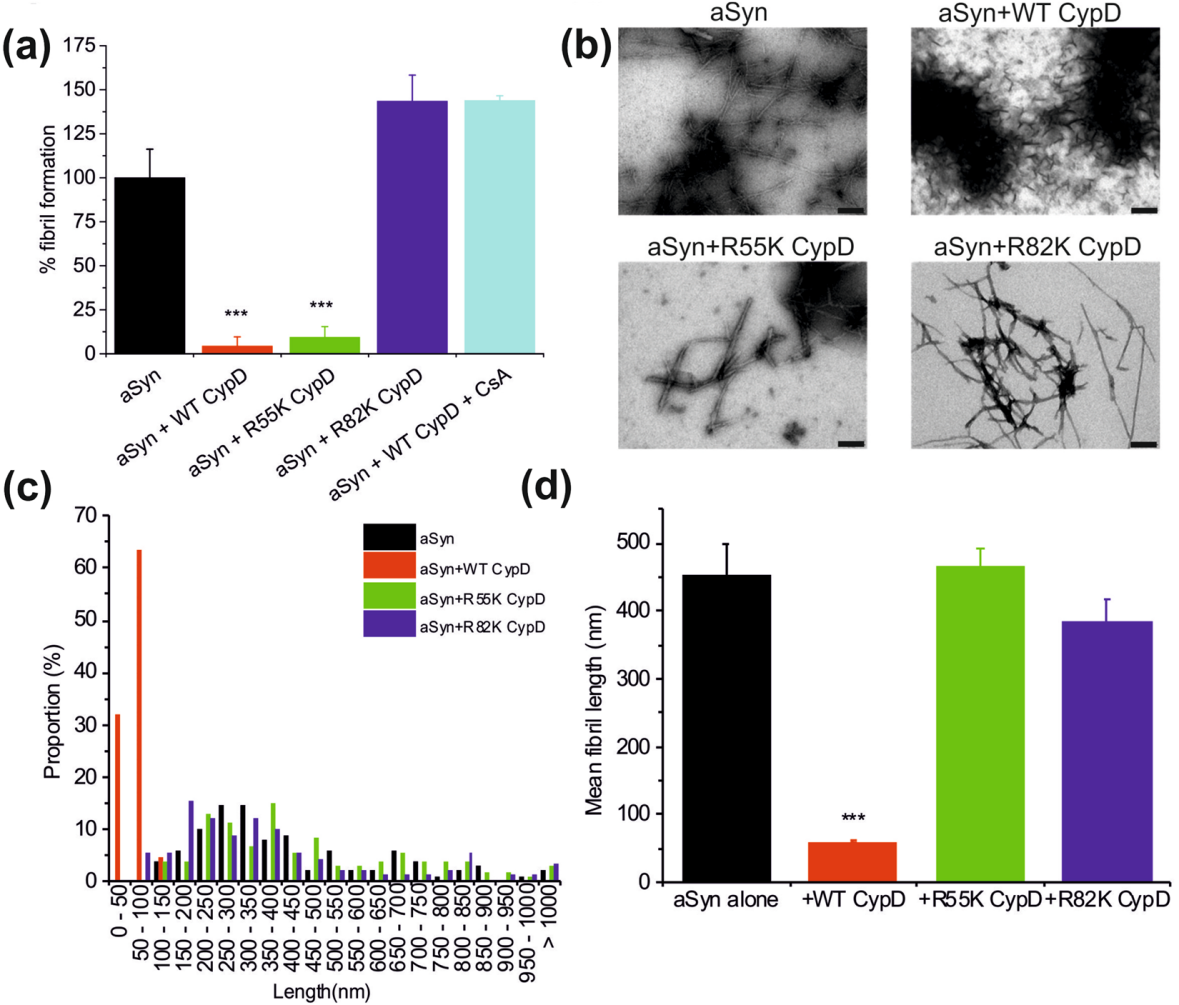
CypD prevents **α**Syn aggregation and disaggregates pre-formed fibrils. (**a**) Thioflavin T fluorescence for αSyn alone and incubated in the presence of CypD WT, R55K and R82K mutants, and CypD WT +CsA in PBS at 37 °C with agitation for 3 days, suggests that CypD and R55K prevents aggregation of αSyn. Control CypD alone is also shown. Data is shown as mean ± SD in triplicate. (**b**) Transmission electron microscope images of αSyn fibrils formed at 37 °C with agitation for 7 days, then further incubated quiescently for 3 days in the presence of CypD WT and catalytically compromised mutants (R55K and R82K). Scale bar is 200 nm. (**c**) Fibril lengths were analysed using ImageJ and shown as proportion of total measurements for each length range. Four images were used for each analysis with 102 ± 8 measurements in total. (**d**) Mean fibril length is reduced when fibrils are incubated with WT CypD suggesting disaggregation. Mutant CypDs do not show disaggregation. ***P < 0.001 by ANOVA with post hoc analysis using Tukey Test carried out in OriginPro 9.

I*n-vitro* experiments using *E.Coli* expressed and purified recombinant proteins revealed that CypD had effects on both αSyn aggregation and fibril stability. Incubation of CypD with freshly prepared αSyn repressed the aggregation of αSyn, as assessed using Thioflavin T (ThT) fluorescence, over a period of 3 days compared with αSyn alone (Fig. 1a). More interestingly, exposure of preformed αSyn fibrils to CypD resulted in a change in fibril morphology, as observed by Transmission Electron Microscopy (TEM) (Fig. 1b). Detailed analyses of the fibril size showed that CypD reduced the lengths of the fibrils. αSyn fibrils were a mixture of fibril sizes of up to >1000 nm in size whereas when incubated with CypD, the particles lengths dropped to less than 100 nm (Fig. 1c,d). CypD alone did not form fibrils. TEM and associated fibril length measurements show that CypD had a disaggregating effect on preformed αSyn fibrils and with a CypD concentration dependency (Supplementary Fig. S1). To distinguish the effects of CypD binding and isomerase activity on αSyn aggregation and disaggregation, the effects the cyclosporine A (CsA), which inhibits both CypD isomerase and binding activities, were tested alongside two CypD mutants: R55K, which has minimal isomerase activity but retains CypD binding to substrate peptides and R85K which has approximately 20% isomerase activity but only binds very weakly to substrate peptides (Supplementary Fig. S5). In the presence of CsA, CypD does not appear to affect αSyn aggregation, confirming CypD’s role in preventing αSyn aggregation. The R55K mutant prevents αSyn aggregation, similar to wild-type CypD (Fig. 1a) but does not promote disaggregation (Fig. 1d). On the other hand, the R82K mutant neither prevents αSyn aggregation nor promotes its disaggregation (Fig. 1a,d). The two mutants does not affect the morphology of αSyn (Fig. 1b) and show a similar length profile as found for αSyn alone (Fig. 1c) with similar mean length fibrils (Fig. 1d).

Taken together the data shows that CypD prevents αSyn aggregation and promotes the disassembly of αSyn fibrils *in vitro*. The data from the R55K and R82K mutants suggest that CypD binding to αSyn is necessary to prevent αSyn aggregation whereas the peptidyl-prolyl isomerisation drives the disaggregation of αSyn fibrils. Cell viability assays showed no alteration to cell viability between mature fibrils and shorter fibrils which were produced by incubating mature fibrils with CypD (Supplementary Fig. S2). This confirms that the breakup of fibrils by CypD does not produce additional toxic αSyn species.

### Disaggregation of αSyn fibrils by Nuclear Magnetic Resonance (NMR)

**Figure 2:**
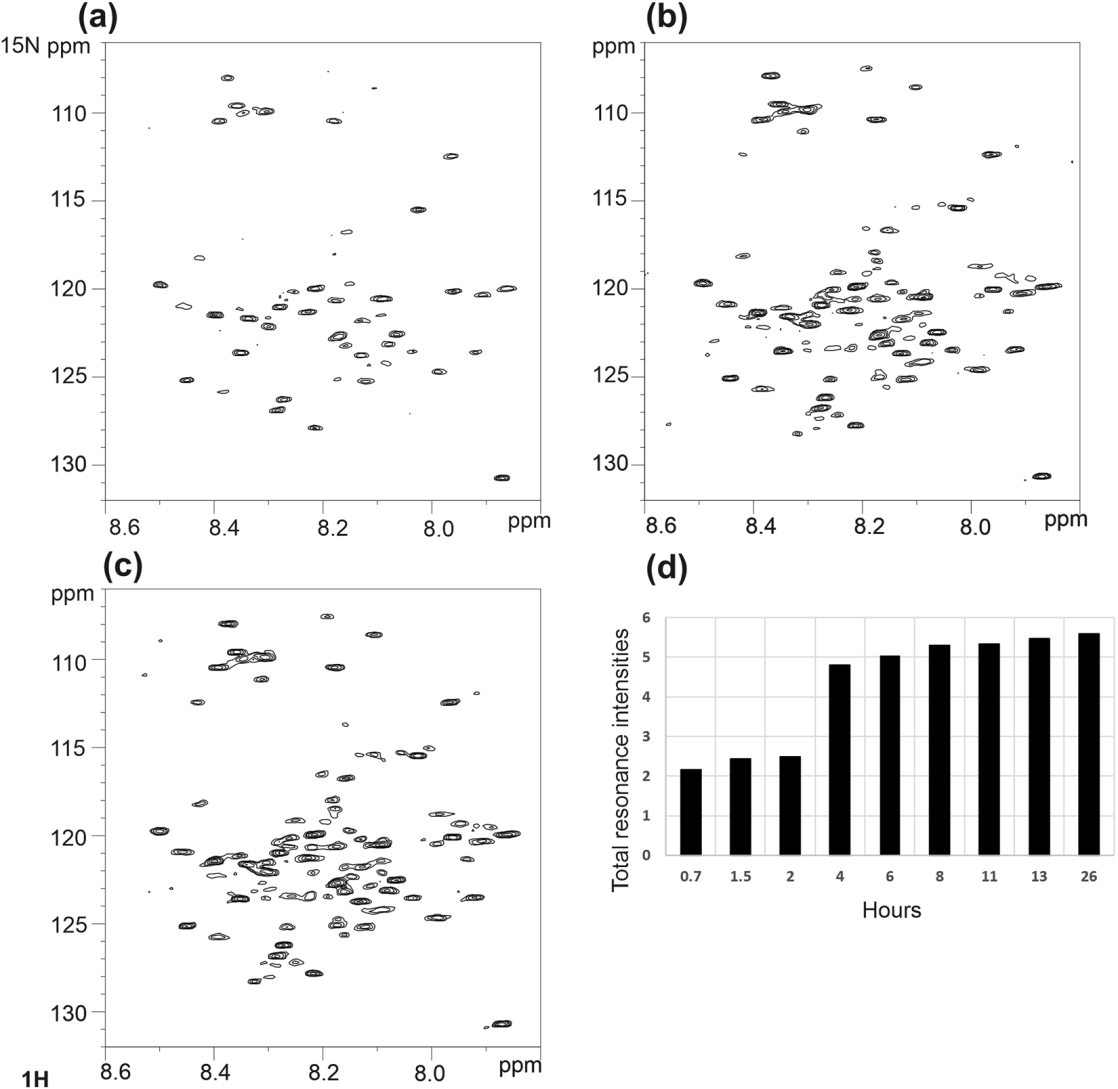
Disaggregation of preformed αSyn fibrils monitored by ^15^N HSQC NMR. Spectra of ^15^N- αSyn fibrils (100μM) in the presence of unlabelled CypD (300 μM) in 20 mM phosphate buffer, 20 mM NaCl, pH 6.5, 298 K at time (**a**) 0 hrs (**b**) 8 hrs, (**c**) 36 hrs. (**d**) Histogram showing increase of αSyn resonance intensities after addition of CypD. The results show a progressive recovery of signal-to-noise of the αSyn resonance with time of CypD incubation suggesting dissolution of the pre-formed fibrils.

A sample of ^15^N-labelled αSyn was agitated for several days to encourage the formation of fibrils and the ^1^H-^15^N Heteronuclear Single Quantum Coherence (HSQC) spectrum acquired (Fig. 2a). The sample was a heterogenous mixture of αSyn forms – monomer, oligomers and fibrils. This explains why many αSyn peaks are observable in the spectrum, particularly residues from the C-terminus region thought to be excluded from the core of αSyn fibrils^19–21^. Notably, however, resonances from residues 37–60 are not observable in this “fibrillar” sample; this region is part of the proposed Greek key structure of αSyn (residues 36–97) where intermolecular hydrogen bonds form the beta-sheet structure of the fibrils^20,21^. Addition of CypD to this “fibrillar” αSyn sample followed by ^1^H-^15^N HSQC data collection over 48 hours period saw a gradual recovery of many αSyn resonances (Fig. 2). This suggests a dynamic process in which increasing amounts of less fibrillar state(s) of αSyn were being formed over time, enabling the resonances of αSyn to be detected. This NMR data agrees with the TEM and ThT data presented in Fig. 1 which points to CypD reducing αSyn aggregation and promoting disassembly of αSyn pre-formed fibrils.

### NMR Studies of αSyn-CypD protein Interactions

**Figure 3:**
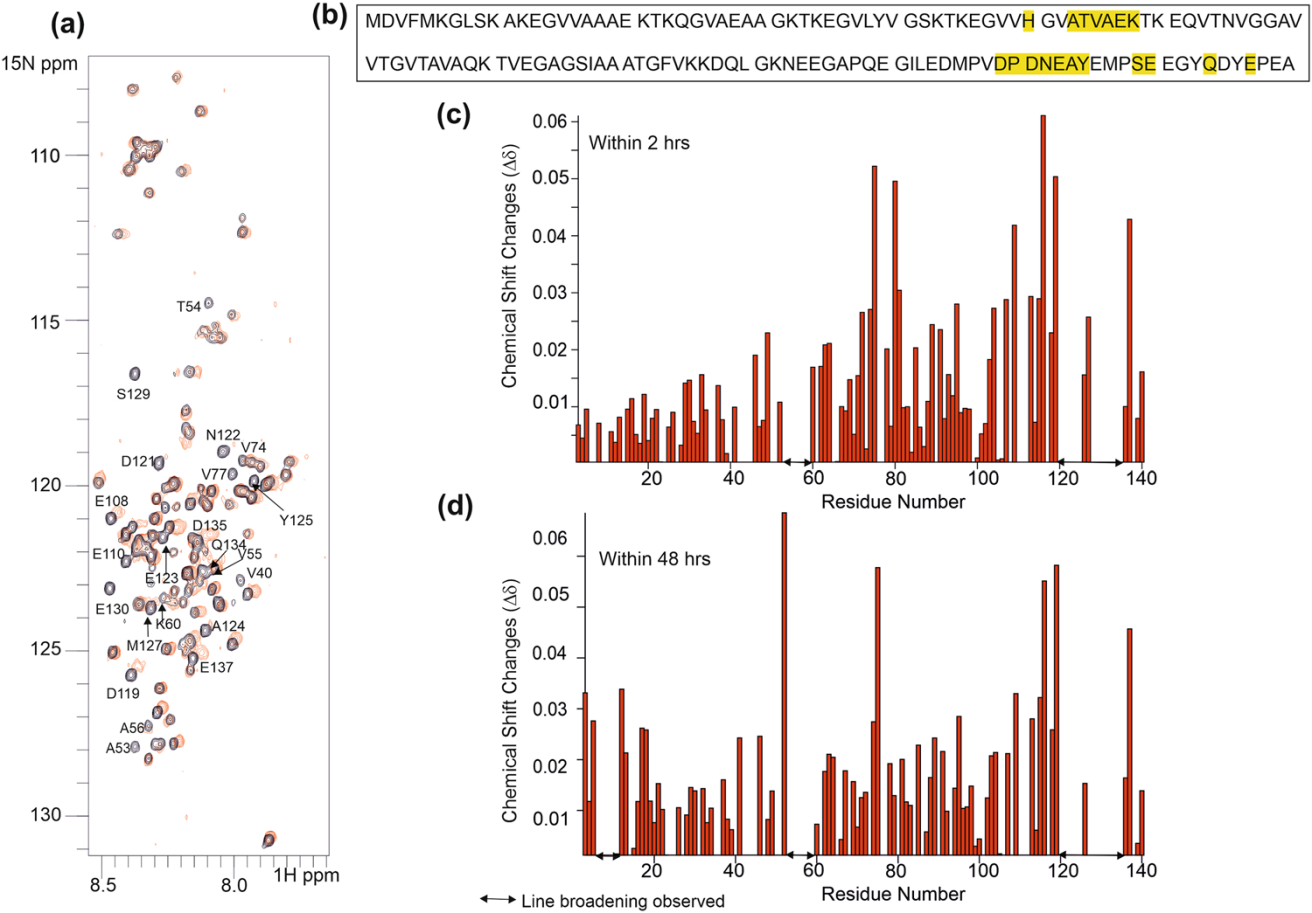
Identification of CypD binding sites on αSyn. (**a**) ^1^H-^15^N HSQC spectra of ^15^N- αSyn (100μM) (black) in the presence of unlabelled CypD (final concentration of 1 mM) (red) in 20 mM phosphate buffer, 20 mM NaCl, pH 6.5, 298 K. (**b**) Amino acid sequence of αSyn with residues whose resolved resonances are significantly broadened shortly after addition of CypD highlighted in yellow. (**c,d**) Histogram of chemical shift changes within 2 hrs (c) and 48 hrs (d). The gaps are from either proline residues or residues with very severely broadened and undetectable at 25 °C; most significantly are the contiguous stretch of early broadened residues from the unstructured C-terminus region 119–135 and residues 50–60.

NMR was used to detect binding and identify the binding site residues from each protein. Despite significant resonance overlap in the spectrum of the unstructured αSyn, over 87% of the non-proline NH resonances are assigned to specific residues (Supplementary Fig. S3). Addition of CypD to ^15^N-labelled αSyn induced selective shifts and line-broadening in the αSyn ^1^H-^15^N HSQC NMR spectra (Fig. 3). The maximum shift changes induced are very small (approximately 0.06) and these are regarded as arising for small changes in solution conditions. The line-broadening effects are better indicators of site of interactions.

Resolved ^1^H-^15^N resonances from predominantly two regions of αSyn residues are significantly broadened in the presence of CypD: H50, A53-K58 (part of the segment known as pre-non-amyloid β component(NAC) region) and E110, D119-Y125, S129, E130, Q134 and E137 (Fig. 3a,b). The resonances of other residues from these regions are likely to experience similar broadening but due to resonance overlap, these cannot be straightforwardly interpreted. Resonances from some residues in the NAC region (residues 60–95) are only minimally shifted and with little line-broadening (T75, V77, K80). The resonance line-broadening is more extensive with time; interestingly some residues at the N-terminus (resolved resonances V3, F4, M5, L8 and S9) show line-broadening after several days, although overall, more than 55% of the αSyn resonances are still observable (Fig. 3c). These results suggest the absence of generalised aggregation of the system, in line with the ThT results (see above). The addition of lysozyme as a negative protein control showed no NMR effects on any of the resonances in the αSyn ^15^N-^1^H HSQC spectrum, confirming that the observed selective signal perturbations are CypD-dependent.

**Figure 4:**
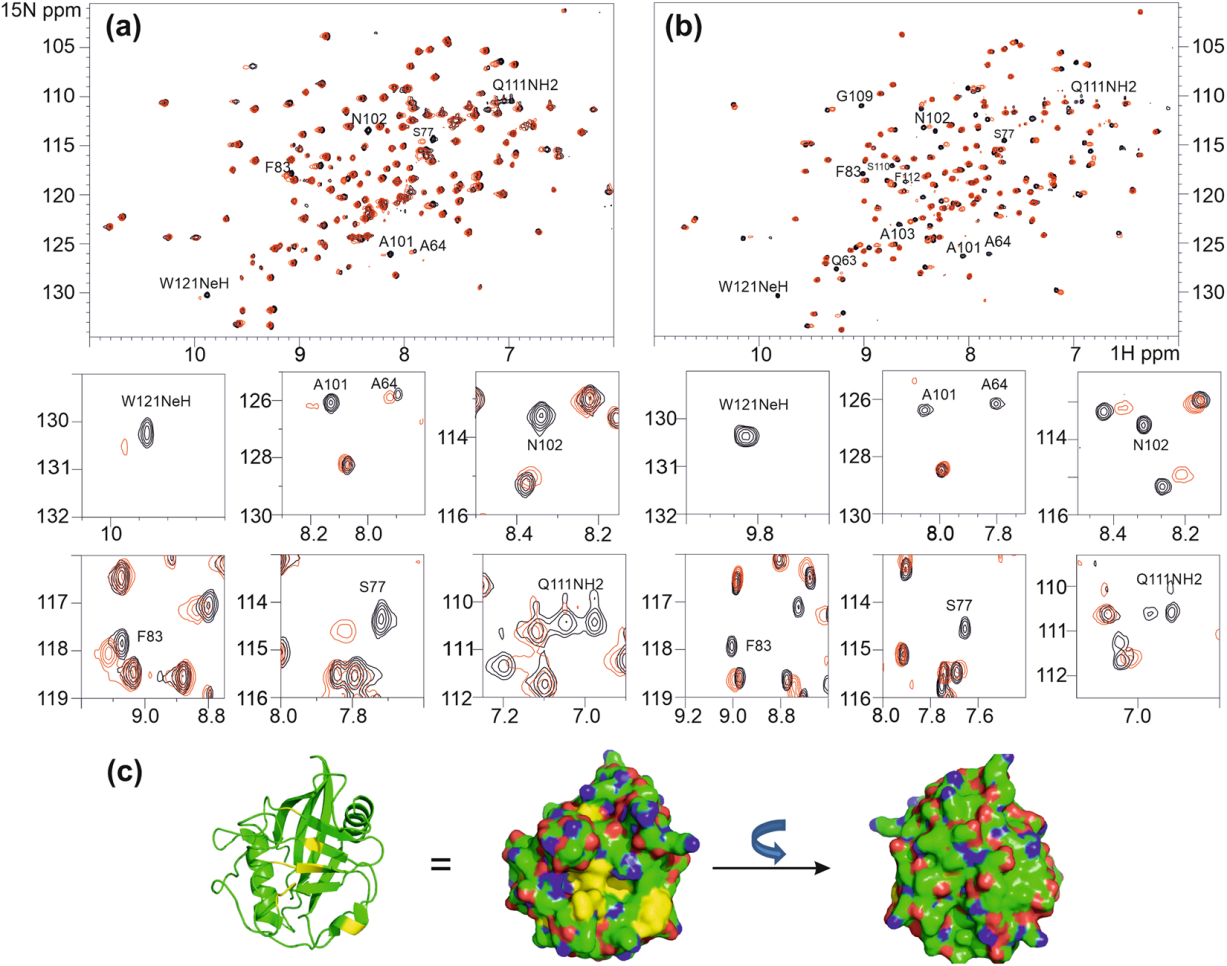
Identification of αSyn binding sites on Cyp D. (**a**) ^1^H-^15^N HSQC spectra of ^15^N-CypD (100μM) (black) in the presence of unlabelled αSyn (final concentration of 1 mM) (red) in 20 mM phosphate buffer, 20 mM NaCl, pH 6.5, 298 K. Expanded plots of the line-broadening of several residues are shown below the main plot. (**b**) ^1^H-^15^N HSQC spectra of ^15^N-CypD (50 μM) (black) in the presence of αSynC peptide (final concentration of 1 mM) (red) in 20 mM phosphate buffer, 20 mM NaCl, pH 6.5, 283 K (the lower temperature was used to increase affinity of the peptide binding to CypD and reduce exchange rate). Expanded plots of the line-broadening of several residues are shown below the main plot. (**c**) Cartoon representation and molecular surface of CypD (PDB 2BIT) with residues that show significant changes in chemical shifts and/or linewidths coloured in yellow. Many of the residues affected by the presence of αSyn are in or surround the putative ligand binding pockets of CypD. The molecular surface is shown in two orientations to demonstrate that residues affected by αSyn are confined to one surface. The structures were created using the program Pymol (The PyMOL Molecular Graphics System, Version 1.3, Schrödinger, LLC).

Reciprocally, selected resonances in ^15^N CypD ^1^H-^15^N HSQC NMR spectra are affected upon addition of unlabelled αSyn (Fig. 4a). The line-broadening effect is due to an intermediate exchange between the free and complexed proteins. The broadened resonances are likely from residues involved in the intermolecular interactions; these include residues Q63 (main and side-chain), S77, F83, A101, N102, A103, Q111 (main and side-chain), S110, F112, F113, W121 (indole NH).

### CypD interactions with αSyn peptides

To further investigate αSyn interactions with CypD, synthetic peptides were used: 51–65 (αSynM, GVATVAEKTKEQVTN) and 119–140 (αSynC, DPDNEAYEMPSEEGYQDYEPEA) both encompassing the two regions identified in the NMR studies above as being involved in or affected by CypD interactions. The peptide 74–95 (VTAVAQKTVEGAGSIAAATGFV) was used as the negative control; it showed no interactions with CypD and was not a peptidyl-prolyl isomerisation substrate. From the isothermal titration calorimetry data, αSynC binds CypD with K_d_ ~ 78 µM, whereas αSynM binds much more weakly (Supplementary Fig. S4). The NMR data also show that αSynM is also not an isomerisation substrate of CypD. In the ^15^N-^1^H HSQC spectrum of CypD in the presence of αSynM (20-fold excess) the positions of the affected CypD resonances are only slightly shifted from the positions in the free protein with minimal broadening observed for selected peaks (data not shown). These NMR characteristics indicate very weak binding, in agreement with the ITC data.

In the presence of αSynC, more discernible chemical shift changes are observed in the ^15^N-^1^H HSQC spectrum of CypD, with many resonances of the bound state easily detected; however, a subset of resonances residues Q63 (main and side-chain), S77, F83, A101, N102, A103, Q111 (main and side-chain), S110, F112, F113, W121 (indole NH) remains unobservable due to an intermediate chemical exchange rate between the free and complexed protein despite using a 20-fold excess of αSynC (Fig. 4b). The residues affected are identical to the ones observed in the CypD complex formed with intact αSyn, confirming that the αSynC sequence forms the segment that binds CypD. All the affected residues on CypD make up the putative ligand binding pocket for proline-containing peptide substrates (Fig. 4c).

**Figure 5:**
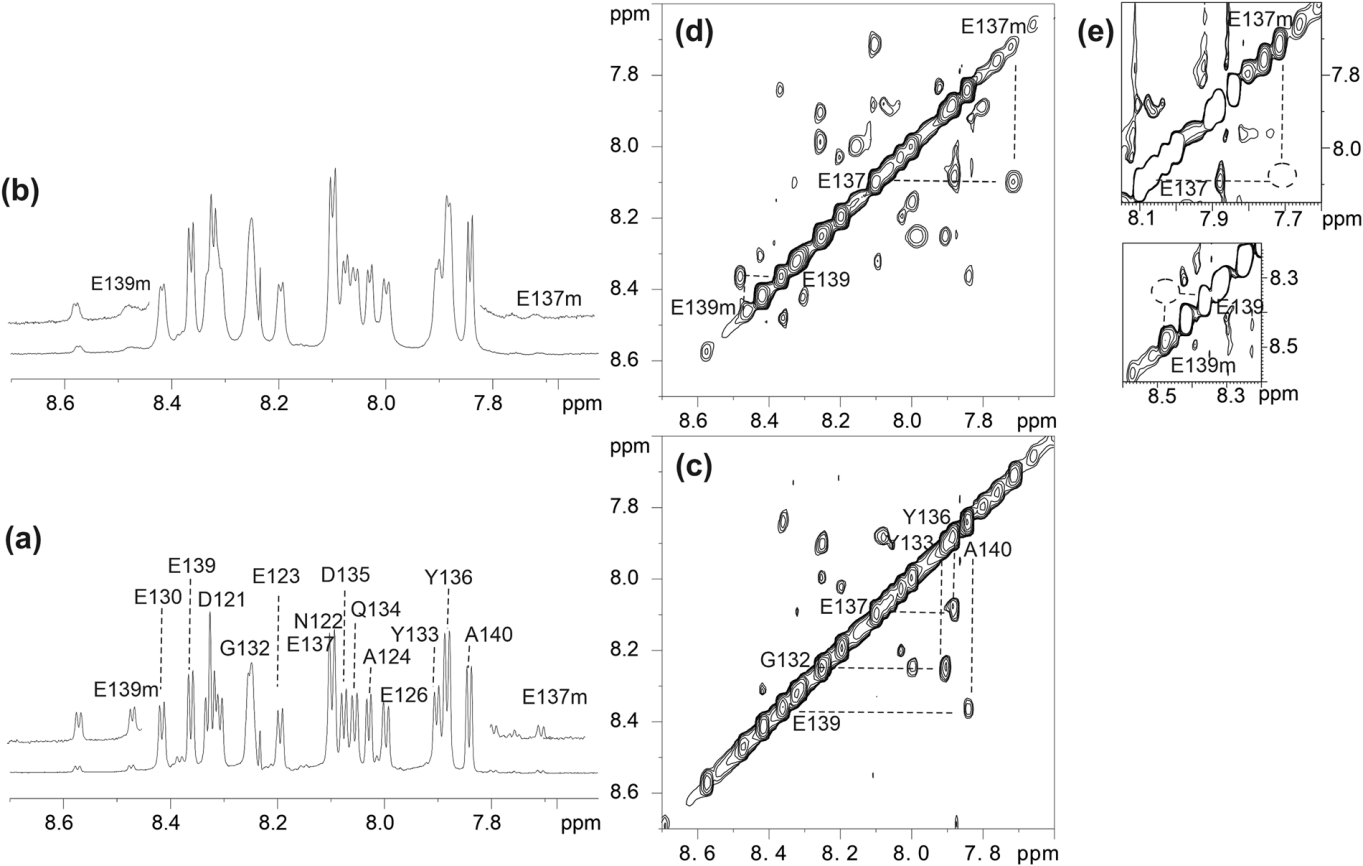
NMR detection of αSynC cis-trans isomerisation by CypD. (**a**) ^1^H spectrum of the amide region of αSynC (1 mM, 283 K) with expansion to show the presence of minor forms (E137 m, E139 m). (**b**) ^1^H spectrum of the amide region of αSynC (1 mM) in the presence of ^13^C, ^15^N CypD (50uM) where broadening of resonances from both major and minor forms are observed (using 1D ^13^C,^15^N-filtered experiment). (**c**) 2D ^1^H-^1^H NOESY spectrum of αSyn C at 283 K, mixing time 350 ms. (**d**) ^13^C, ^15^N-filtered 2D NOESY (283 K, mixing time 350 ms) spectrum of αSyn C (1 mM) in the presence of ^13^C, ^15^N CypD (50uM) shows the presence of chemical exchange cross-peaks between the major (E137 and E139) and minor (E137m and E139m) forms of the peptide. (**e**) Same sample as (**d**) in the presence of 100uM Cyclosporine A which shows that the disappearance of the exchange cross-peaks due to the inhibition of CypD isomerase activity.

The proton spectra of αSynC alone reveal that more than one set of peaks for each amide group is present (Fig. 5a). This is due to the presence of *cis* and *trans* peptidyl-prolyl isomers; peak integration estimates a 9:1 *trans:cis* ratio. Addition of a small amount of CypD (1/20^th^ of peptide concentration) induced selective line-broadening effects, as shown for the resolved amide ^1^H resonances from E123, E130, Y133, D135, Y136 (Fig. 5b); the resonances of the minor conformers are significantly broadened to the extent that their peaks are hardly observable. However, D121, N122, E139, A140 amide groups, which are residues at either end of αSynC, are minimally affected by the presence of CypD. Hence, of the three peptidyl-prolyl bonds present in αSynC (involving P120, P128 and P138), it is appears that the one involving P128 is the most likely isomerisation site of CypD.

NH-NH NOESY cross-peaks are observed between amide resonances of the major polypeptide chain in the 2D NOESY spectrum of αSynC at long mixing times (350 ms) (Fig. 5c). Upon the addition of a sub-stoichiometric amount of CypD, extra cross-peaks are observed which are attributed to chemical exchange between the major and minor forms (Fig. 5d). This shows that the rate of isomerisation between the major *trans* conformation and the minor *cis* form is increased by the isomerase activity of CypD. The exchange crosspeaks disappear upon addition of CsA to the complex (Fig. 5e) or when using the R55K CypD mutant which has minimal isomerase activity (Supplementary Fig. S5) but is still able to bind with a similar affinity as the wild-type protein (Supplementary Fig. S5). These NMR data provide strong evidence that αSynC is an isomerase substrate of CypD.

We conclude from both the ITC and NMR studies that CypD binds to the acidic C-terminal region of αSyn and most likely isomerises the M127-P128 bond found in this region, similar to the data obtained for the αSyn-CypA interactions^22^.

## Discussion

A range of proteins and small molecules have been shown to either promote or prevent αSyn aggregation, as well as dissociate preformed fibrils. An emerging picture is that agents that prevent aggregation can also dissemble amyloid fibrils. Some of these examples include: the trimeric chaperone system of Hsc70, Hsp110 and J-protein which together disaggregate αSyn fibrils in an ATP-dependent manner^14^; the small molecule promethazine prevents the aggregation of human insulin and human lysozyme, and depolymerises preformed fibrils^23^; epigallocatechin-3-gallate (EGCG) dissociates αSyn aggregates^24^; and memantine inhibits Αβ aggregation and disassembles preformed Aβ fibrils^25^.

While the aggregation processes are well studied the same is not true for disaggregation. Some details are beginning to emerge, in particular from studies of the polyphenolic disaggregases such as EGCG which binds to the C-terminus of monomeric αSyn^26^. αSyn forms fibrils when left in buffer *in vitro*; this is a dynamic process, with the fibrils in equilibrium with the monomeric state. EGCG binding to the C-terminus stabilises the αSyn monomeric state and shifts the monomer-fibril equilibrium towards the less aggregated forms. The data reported here which shows CypD interacting with αSyn C-terminus fits this model. More interestingly, we demonstrate that while CypD binding is sufficient to prevent αSyn aggregation, increased peptidyl-prolyl isomerisation rate is required for disaggregation of the preformed αSyn fibrils. Cell viability assays appear to show no deleterious effects on cell survival when CypD was present suggesting that the shortened fibrils produced do not display enhanced toxicity although more extensive work is required.

The involvement of immunophilins in neurodegenerative diseases has been known for some time although molecular mechanisms are largely unknown^27^. Cyclophilins have previously been shown to either reduce the oligomerisation of amyloidogenic proteins or to cause fibrils to disassemble. Human Cyp40 is a multidomain protein consisting of the catalytic isomerase domain followed by four tetratricopeptide repeats. Through mutagenesis, the isomerase domain was identified as being responsible for this disaggregating activity^15^ as is also shown here for CypD. CypD also interacts directly with the Alzheimer’s Disease Aβ peptide and this interaction, *in vitro*, suppressed fibril formation of the peptide Aβ(1–40)^28^. Cyclophilin A has recently been shown to bind to αSyn, also at the C-terminus as well as to the preNAC sequence, similar to the results obtained here^22^. Another peptidyl-prolyl isomerase immunophilin protein belonging to the FKBP family, cytoplasmic FKBP12, has also been shown to bind αSyn^29^. Unlike the results here, FKBP 12-binding promoted αSyn aggregation^29,30^. However, the failure to find appropriate FKBP inhibitors, apart from FK506, to prevent αSyn aggregation, or that other FKBPs do not seem to be such potent promoters of αSyn aggregation as FKBP12, means that more work is required to unravel the complexities of the role of FKBPs in αSyn aggregation^31^.

The present study adds mitochondrial CypD to the list of agents that can promote αSyn disaggregation, this occurring through binding and isomerisation of the unstructured, negatively charged, proline rich C-terminus region. CypD is a peptidyl-prolyl isomerase and is known to bind to specific consensus motifs which contain a proline residue. As there are no proline residues in the H50-N65 segment, and the isothermal titration calorimetry (ITC) data showed extremely weak interactions between a peptide corresponding to this segment and CypD, this region is unlikely to be the CypD binding site. The significant NMR resonance broadening observed in this central region of full-length αSyn when CypD is present is possibly due to conformational changes resulting from CypD interacting with the C-terminal region. Furthermore, the A53T mutant appears to bind CypD with similar NMR shift characteristics, providing further evidence that this region is not the primary CypD-binding site since mutation from A to T would be expected to make a difference to binding (Supplementary Fig. S6)

αSyn is natively unfolded under physiological conditions^32^. During aggregation, the αSyn structure transitions from unfolded to helical conformation (in association with lipids) to beta-sheet conformation which facilitates intermolecular hydrogen bonding and favours aggregation; the N-terminal residue 1–60 polypeptide chain has seven repeats bearing the KTKEGV motif and this region can be induced to adopt helical conformations in the presence of lipids^33–35^. The middle segment (amino acid 61–95) is a hydrophobic region with a high propensity for β-sheet secondary structure; it is this region which forms the nucleus for amyloid formation^34,36^. The acidic, negatively charged C-terminus segment (96–140 residues) remains highly disordered in all the currently identified different states of αSyn^34^. αSyn progression from an unfolded monomer to fibrils takes several routes and it is hypothesised that many different conformations are adopted which are in equilibrium with one another. It has been difficult to develop therapeutic agents due to this structural heterogeneity and dynamics. However, molecules like CypD, appear to be capable of binding to one form with sufficient affinity to shift the monomer-multimer/fibril equilibrium in favour of the monomeric state, reducing the likelihood of pathogenic forms accumulating; this characteristic could present new opportunities to the design of therapies against amyloid diseases.

Protein disaggregation can be viewed as advantageous, being a housekeeping process that helps maintain proteostatis, and prevents the cell from accumulating persistant oligomer, aggregates and fibrils. However, disaggregation produces small oligomeric species, and since the relative toxicity of amyloid fibrils and smaller oligomeric species is still debated, it is necessary to be cautious before concluding that the disaggregation has a positive effect; the smaller species resulting from disaggregation could in fact provide the “seeds” for further aggregation^37^. Hence, although the ability of CypD to prevent αSyn is clearly beneficial, the same cannot yet be concluded for its role as a disaggregase.

This is one of the most detailed molecular investigations involving CypD and a partner protein in that it (i) identifies CypD binding sites on target proteins, (ii) measures the binding affinities between CypD and peptides of the intact partner protein, (iii) investigates the link between CypD binding and isomerisation, (iv) investigates the effects on the structure of the target protein when CypD binds, and (v) uses *in vitro* measurements to demonstrate that interaction with CypD results in reduced fibrillation of a physiologically-validated target protein. Consequently, the results here provide some insights into how CypD reduces protein aggregation and aggregate accumulation in the mitochondria. Whether this role of CypD has positive cellular effects will need to be investigated further.

## Methods

### Peptide synthesis

The synthetic peptides αSynM (residues 50–65, GVATVAEKTKEQVTN) and αSynC, residues 119–140, DPDNEAYEMPSEEGYQDYEPE140A) were purchased from Gemini Biosciences, UK and delivered >98% pure. Control peptide αSyn 74–95 (VTAVAQKTVEGAGSIAAATGFV) was purchased from Protein Peptide Research Ltd, UK and delivered >98% pure.

### CypD expression and purification

Recombinant CypD was produced as the mitochondrial mature form corresponding to residues 43–207 of the fully translated gene sequence (Uniprot acc. P30405). The gene for the mature protein was cloned in pETM11 vector (EMBL). R55K and R82K point mutations were introduced into the CypD gene using Agilent QuikChange II XL site-directed mutagenesis kit (#200521), using the plasmid pETM11 CypD as template. Mutagenesis was carried out as described in the protocol supplied with the kit. The following oligonucleotides and their complementary equivalents were used: R55K forward and reverse oligonucleotides, respectively: 5′GCTCCACCTTCCACAAGGTGATCCCTTCCTT-3′ and 3′-AAGGAAGGGATCACCTTGT GGAAGGTGGAGC-5′; R82K forward and reverse oligonucleotides, respectively: 5′-TCCATCTACGGAAGCAAGTTTCCTGACGAGAAC-3′ and 3′-GTTCTCGTCAGGAAACTTGCTTCCGTAGATGGA-5′. Wild-type and mutant proteins were expressed and purified in the same way. Proteins were expressed in BL21 (DE3), Novagen) *E. coli* cells which were grown either in LB or 2xM9 minimal media (^15^NH_4_Cl (1 gm/litre) or ^15^NH_4_Cl/ ^13^C_6_-Glucose (4 gm/litre) (in the case of deuterated ^15^N samples, ^15^NH_4_Cl together with D2O, instead of H_2_O, were used)) at 37 °C to OD600 nm ~0.7 and induced with 1 mM isopropyl β-D-1-thiogalactopyranoside (IPTG), incubated at 18 °C, and harvested 16 hours later (4 °C, 4500 × g). The cell pellet was resuspended in 50 mM Tris-HCl pH 8, 500 mM NaCl, 10% glycerol pH 8 (Buffer A), and lysed using pressure homogenization. The cellular debris and insoluble fraction were removed by centrifugation (4 °C, 32,000 × g). The soluble crude lysate fraction was passed through a 0.4 micron filter prior to loading onto a Buffer A pre-equilibrated 5 mL HisTrap column. CypD was eluted from the column against an increasing imidazole gradient (Buffer A + 500 mM imidazole). Fractions containing CypD were pooled and dialysed against fresh Buffer A. TEV protease was then added to the sample at a ratio of 1:20 TEV protease:Protein and incubated overnight at room temperature with gentle rotation. The cleaved His tag and remaining contaminating proteins were removed by reapplying to the HisTrap column, whereby the untagged CypD was eluted in the flow through and collected. The CypD solution was further purified via size exclusion chromatography, using a Superdex 75 column in 50 mM Na phosphate pH 6.8, 100 mM NaCl, 5 mM DTT, 2 mM EDTA. The recombinant protein was verified by mass spectrometry.

### αSyn expression, purification and formation of fibrils

Wild-type αSyn was expressed from a pRK172 plasmid kindly provided by Michael Goedert (LMB, Cambridge). The expression construct was transformed into chemically competent LEMO cells. Expression cultures were prepared in minimal growth medium and grown at 37° to OD600 nm ~ 0.8 and induced with 0.5 mM. The cultures were incubated at 18 °C with shaking at 180 rpm overnight. Cells were harvested through centrifugation at 4,000 g for 20 mins at 4 °C. Cell pellets were snap frozen in LN_2_ prior to storage at −80 °C.

Cell pellets were thawed and resuspended in 20 mL Buffer A (20 mM Tris-HCl pH 8.0, 1 mM EDTA). To each resuspended pellet a single Complete™ protease inhibitor tablet (Roche, Switzerland) was added. The cells were lysed via pressure homogenization and the lysate was stored on ice. DNA/RNA was removed by the addition of benzonase (Sigma-Aldrich, USA). The lysate was then incubated at ~85 °C for 10 mins, after which the lysate was clarified by centrifugation at 18,000 g for 30 mins at 4 °C. The clarified lysate was then loaded onto a 5 mL HiTrap Q HP column pre-equilibrated with Buffer A. The protein was removed by a gradient elution with Buffer B (Buffer A +1 M NaCl). Fractions were analysed by SDS-PAGE. The fractions containing αSyn were pooled and applied to an Amicon Ultracentrifuge (Merck Millipore, Germany) filter unit with a 30 kDa MWCO. The flow-through was collected and then applied to an Amicon Ultracentrifuge filter unit with a 10 kDa MWCO. The material was concentrated and the concentration was determined by absorbance at 280 nm (MW = 14.5 kDa and ε = 5600 M^−1^ cm^−1^). Purity was assessed by SDS-PAGE and mass spectrometry. Purified protein was dialysed into H_2_O and lyophilized for long-term storage.

To make αSyn fibrils, lyophilized αSyn was resuspended in hexafluoroisopropanol (HFIP) and vortexed for 30 s. The HFIP was evaporated off under a stream of N2 and the process repeated three times. The protein film was resuspended in freshly prepared phosphate buffer saline (PBS) pH 7.4 such that the protein concentration was 100 µM. The protein was incubated at 37 °C with agitation for 1 week.

### Thioflavin T fluorescence (ThT)

αSyn was incubated at 50 μM alone or in the presence of 3-fold molar excess of CypD (WT, R55K and R82K mutants and pre-formed CypD-CsA complex) with agitation using a flea in PBS at 37 °C for 3 days. αSyn was diluted to 20 µM and fibril formation assessed using the fluorescent dye ThT (added at final concentration of 20 μM) in 100 μL. Fluorescence measurements were carried out in triplicate using a Flexstation 3 microplate reader (Molecular Devices Ltd., USA) in 96-well black-walled, clear-bottomed microplates, with excitation at 450 nm and emission at 485 nm.

### Transmission electron microscopy

Disaggregation was probed by incubating 50 µM of pre-formed fibrils in the presence of 3-fold molar excess of CypD (WT, R55K and R82K mutants) at 37 °C for 3 days without agitation. Morphologies of the insoluble aggregates were analysed using negative staining methods (4% uranyl acetate). Peptide suspensions (10 μL) were loaded onto carbon coated copper grids and visualised on a Tecnai 10 electron microscope at 120 kV. Images were analysed for fibril length using ImageJ with the scale bar as the reference. Four images were used for each analysis with 102 ± 8 measurements in total.

### Nuclear magnetic resonance (NMR) spectroscopy

NMR spectra were acquired on Bruker Avance III 800 MHz spectrometer equipped with [^1^H, ^15^N, ^13^C]-cryoprobes. 1D and 2D spectra of αSyn peptide were performed using 1 mM peptide dissolved in 20 mM phosphate, 20 mM NaCl pH 6.5 at 298 K or 283 K. Resonance assignments of the proton spectra of αSyn peptide were obtained using ^1^H-^1^H 2D TOCSY (mixing time 60 ms) and NOESY spectra (mixing time 350 ms). To make the assignments of bound αSynC and to observe the chemical exchange cross-peaks between the major and minor forms of αSynC (at 1 mM) in the presence of CypD (peptide: protein ratio 20:1), ^13^C/^15^N CypD samples were used together with ^13^C/^15^N-filtered 1D, 2D TOCSY and 2D NOESY experiments (350 ms mixing time). ^1^H-^15^N Heteronuclear single quantum coherence (HSQC) experiments for ligand binding screening were performed using 0.1 mM ^15^N-labelled CypD in 20 mM phosphate, 20 mM NaCl pH 6.5, 298 K or 283 K in the absence or presence of αSyn or αSyn peptides. The same buffer was used for obtaining the ^1^H-^15^N spectra of ^15^N-labelled αSyn at 298 K. Experiments using ^15^N-labelled αSyn fibrils were performed at 288 K. ^2^H/^15^N-labelled CypD was used to make the complex with intact αSyn in order to achieve the optimal conditions to observe shift perturbations, taking into account the size of the complex (~32kD). Backbone ^1^H and ^15^N resonances of CypD and αSyn were assigned using standard triple resonance to those assigned by ^13^C, ^15^N, ^1^H-triple resonance HNCA, HN(CO)CA, HNCO, HN(CA)CO, CBCA(CO)NH, and CBCANH experiments.

In the analyses of the chemical shift perturbation, the chemical-shift difference was expressed as: Δδ = {(ΔH)^2^ + (0.15ΔN)^2^}^1/2^. Values of Δδ greater than 0.07 were considered significant.

## Supplementary information

Supplementary information.
