# Supplementary material for: Cyclophilin D binds to the acidic C-terminus region of α-Synuclein and affects its aggregation characteristics
Source: Sci Rep. 2020 Jun 23;10:10159. doi: 10.1038/s41598-020-66200-9 (PMC7311461; doi:10.1038/s41598-020-66200-9)
Supplement: Supplementary file 1 — Supplementary information. [file 41598_2020_66200_MOESM1_ESM.docx]

Cyclophilin D binds to the acidic C-terminus region of α-Synuclein and affects its aggregation characteristics

James Torpey, Jillian Madine, Amy Wood and Lu-Yun Lian

NMR Centre for Structural Biology, Institute of Systems, Molecular and Integrative Biology, University of Liverpool, Liverpool L69 7ZB.

**SUPPLEMENTARY INFORMATION**

**SupplementaryMethods**

Cell Viability Assay

SH-SY5Y cells were grown in T25 flasks in F-12 Ham’s growth medium (supplemented with 10% FBS, 1% Pen-Strep and 1x MEM Essential Amino Acids) and incubated at 37°C 5% CO2. At 90% confluency the cells were washed with PBS pH 7.4 and trypsinized. Trypsinized cells were resuspended in the complete growth medium and plated out onto a black 96-well clear-bottom plate, with 5000 cells/well in a volume of 100µL. 10µL of samples were added to each well. The samples were as follows: Live control (PBS alone), Dead control (1% Triton X-100), 50µM WT αSyn fibrils alone, 150µM WT CypD alone, and 50µM WT αSyn fibrils plus 150µM WT CypD. All samples had been quiescently incubated at 37°C for 3days prior to use. The challenged cells were incubated for a further 24hours. 10µL of cell counting kit-8 (CCK-8) reagent was added to each well. The plate was incubated for a further 4hours and the absorbance at 450nm was recorded every hour using a Flexstation 3 microplate reader (Molecular Devices). The absorbance contribution from phenol red in the cell growth medium was subtracted. The percentage cell viability was calculated based on the absorbance relative to those of the live and dead controls, which represented 100% and 0% viability respectively.

Isothermal Titration Calorimetry (ITC)

All ITC experiments were carried out at 10°C on an ITC200 Microcalorimeter (GE Healthcare). The buffer used was 20 mM phosphate, 20 mM NaCl at pH 6.5. Each experiment consisted of an initial injection of 0.5 µL, followed by fifteen 2.39 µL injections before a final injection of 1.89 µL. Control experiments were performed whereby each compound was titrated into buffer and buffer titrated into CypD. In both cases no heat exchange was detected, confirming that there was appropriate match of buffer conditions with no indication of dilution effects. The titration experiments were performed in duplicates, with 50-100 µM CypD in the cell and 500-2000 µM peptides in the syringe. For some of the titrations, data from two runs were concatenated to achieve a saturating isotherm. All data was analysed using the Origin®7 (OriginLab, Northampton, MA) software programme.

Peptidyl-Prolyl Isomerase Assay.

The chymotrypsin-coupled assay method was used to determine the activity of CypD. The reaction was performed in 50 mM HEPES pH7.5, 25 mM NaCl at 10°C, using a total volume of 250μL for each reaction in disposable micro cuvettes. A 2nM CypD solution in the buffer was first equilibrated at 10°C. A freshly prepared room temperature stock of 15 mg mL^–1^ (approx. 0.50mM) alpha-chymotrypsin in 1mM HCl was added to CypD sample to give a final reaction concentration of 50μM before absorption data collection at 390nm was initiated. The peptide substrate Suc-AAPF-pNA from a 10mM stock of the peptide dissolved in 0.47M LiCl/TFE was then immediately added to give a final concentration of 60μM. The final reaction was monitored for 2.5–5 mins. Data collection and exponential fitting of the absorbance reaction curve after initial mixing were performed on a Varian Cary 300 Bio UV-Vis Spectrophotometer.

Supplementary Figures


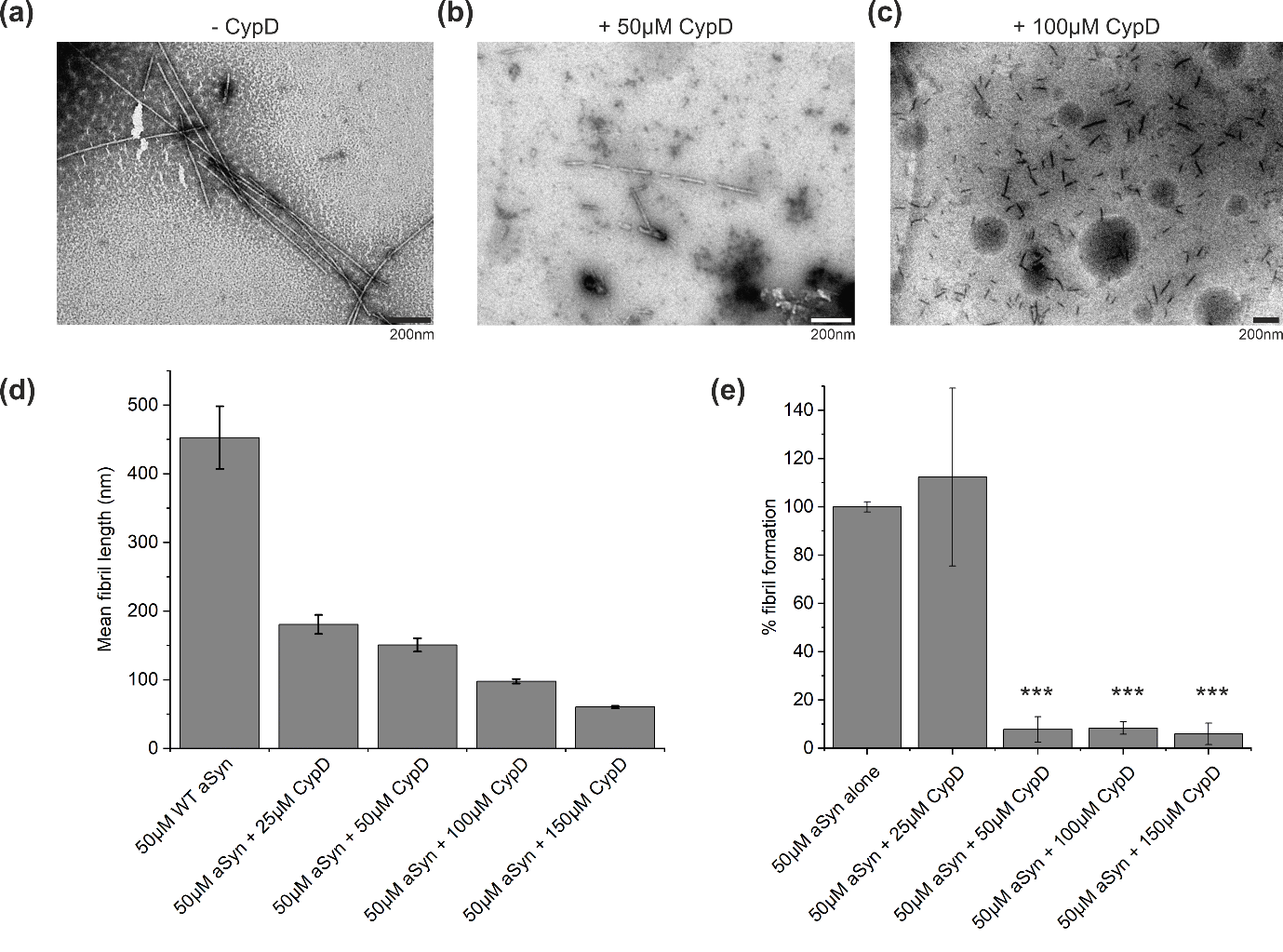


**Figure S1**: Transmission Electron Microscopy (TEM) images and fibril lengths analysis showing the effect of CypD concentration on its ability to disaggregate αSyn fibrils. All experiments were performed using the wild-type CypD and αSyn. TEM images of (a) 50μM αSyn alone, (b) 50μM αSyn plus 50μM CypD, and (c) 50μM WT αSyn plus 100μM CypD. The fibrils were formed by incubation of 50μM aSyn for 7 days with agitation at 37°C, alone and with CypD, followed by further incubation for 7 days at the same temperature without agitation. All images were collected on carbon-coated copper grids and visualized by negatively-staining with 4% uranyl acetate. (d) Histogram showing the fibril lengths data of αSyn alone and with 25-150μM CypD; the number of measurements for each length analysis are given in parenthesis: αSyn alone (103), plus 25μM CypD (52), 50μM CypD (116), 100μM CypD (145), and 150μM CypD (156). (e) Histogram showing the percentage of αSyn fibril found at the different concentrations of CypD.





Figure S2: Cell viability of SH-SY5Y cells challenged with 50µM αSyn fibrils alone (AS), 150µM CypD alone, or 50µM αSyn fibrils that had been disaggregated by 150µM CypD (AS + CypD), as compared to live and dead control samples. Shortening of fibrils may result in an increase in toxicity due to increased surface area. However, the treatment of αSyn fibrils with CypD did not appear to result in increased toxicity.

**
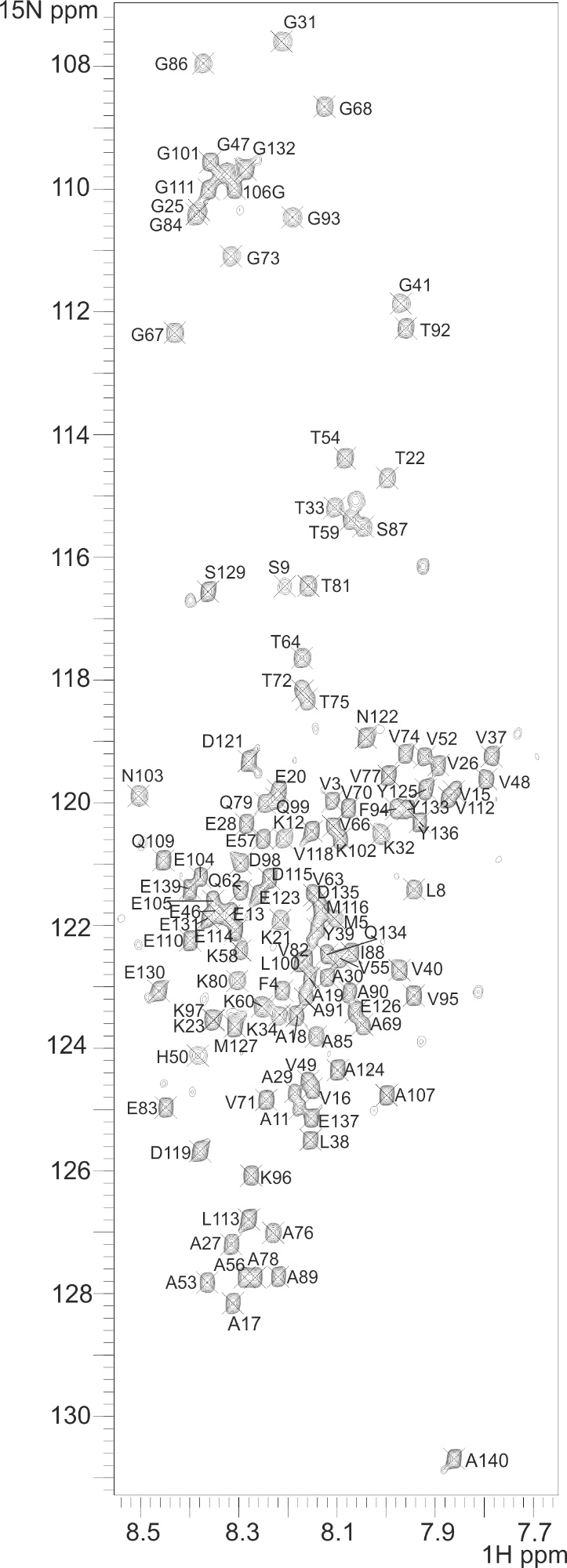
**

**Figure S3:** ^1^H,^15^N HSQC spectrum of αSyn in 20mM phosphate buffer, 20mM NaCl, pH 6.5, 298K acquired on Avance Bruker 800MHz spectrometer with assigned resonances labelled.


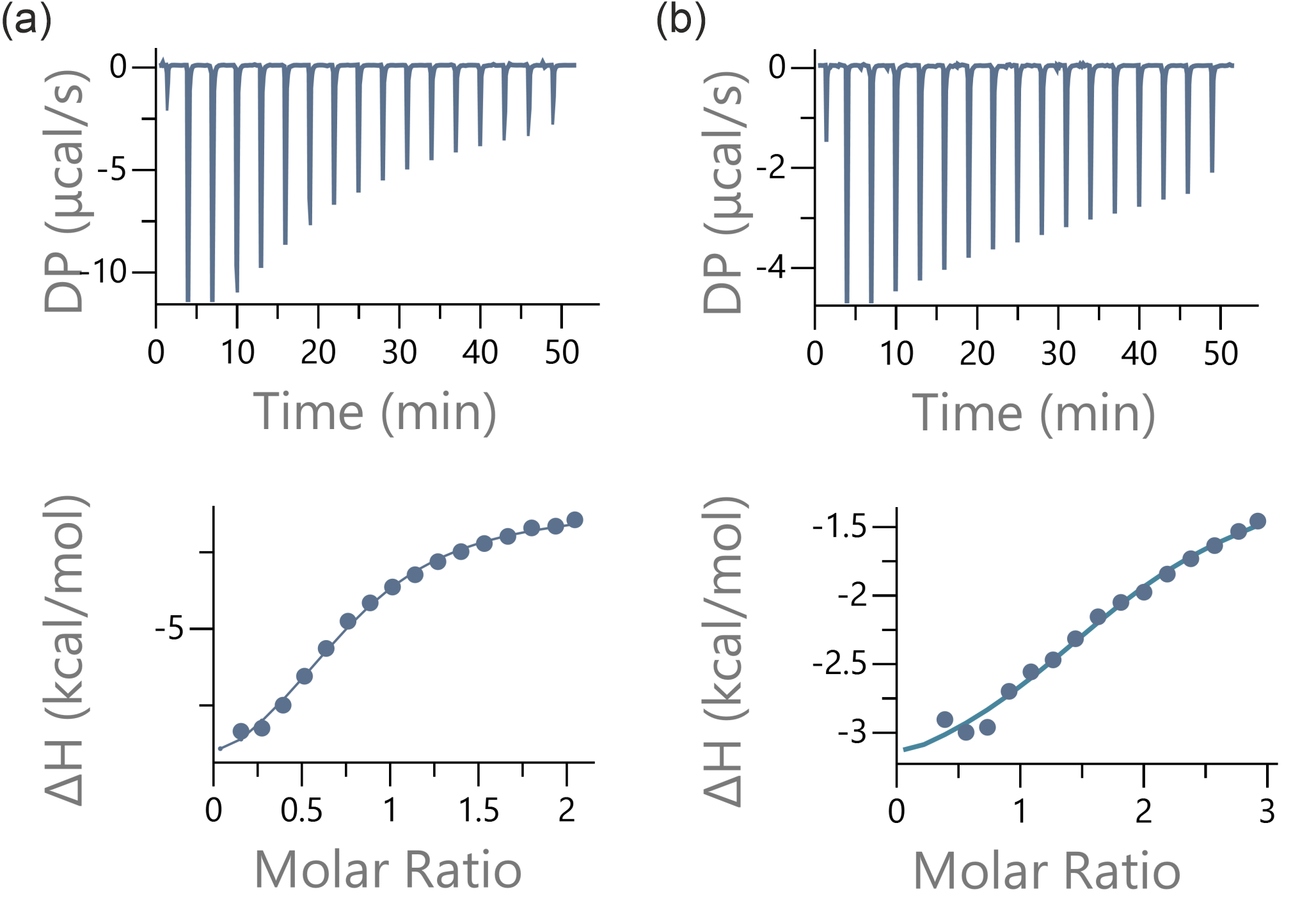


**Figure S4**: Isothermal Titration Calorimetry profiles for the binding of (A) αSynC and (B) αSynM to CypD. The top panel shows the raw calorimetric data obtained upon titration of CypD with each peptide; the bottom panel shows the plots of the integrated heat signal as a function of molar ratio of ligand to protein. The data fit to a one-site binding yielded the K_d_, ΔH and ΔS parameters: K_d_ (αSynC) =78­+10µM, ΔH=-8.85+0.24kcal/mol, TΔS=3.5 kcal/mol/deg. The binding to (αSynM) is too weak to yield a reliable binding isotherm.

**
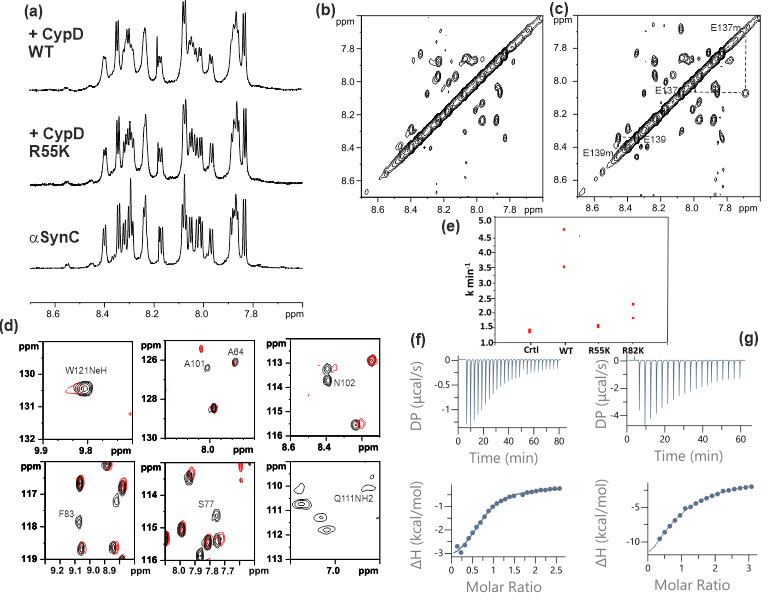
**

**Figure S5**: CypD R55K has reduced isomerase activity when compared to wild-type CypD but is able to bind to αSynC, whereas CypD R82K has both reduced isomerase and binding capabilities, 20mM phosphate buffer, 20mM NaCl, pH 6.5, 283K. (a) 800 MHz ^1^H NMR spectrum of the amide region of αSynC alone (1mM) (bottom), in the presence of CypD R55K mutant (50μM) (middle), and CypD wild-type (50μM) (top); selective line-broadening is detected in the presence of CypD wild-type. (b) 2D ^1^H-^1^H NOESY spectrum of αSyn C at 283K, mixing time 350ms in the presence of R55K CypD (50μM). (c) Same as (b) but with wild-type CypD (50μM) added to the sample in (b), showing the presence of the chemical exchange cross-peaks (indicated by dash lines) between the major (E137 and E139) and minor (E137m and E139m) forms of the peptide. (d) Expanded plots of selected peaks from ^1^H-^15^N HSQC spectra of ^15^N-R55K CypD (50μM) (black) in the presence of αSynC peptide (final concentration of 1mM) (red) to show that R55K can bind to CypD. (e) Comparison of the catalytic rates of Suc-AAFP-pNA isomerisation in the absence of protein (Ctrl) and presence of CypD wild-type, R55K and R82K mutants. (f, g)) Isothermal titration calorimetry profiles, fit to one-site binding, of αSynC interactions with (f) R55K, K_d_ (αSynC) ~68+2µM and (g) R82K, K_d_ (αSynC) ~153 + 12uM.

**
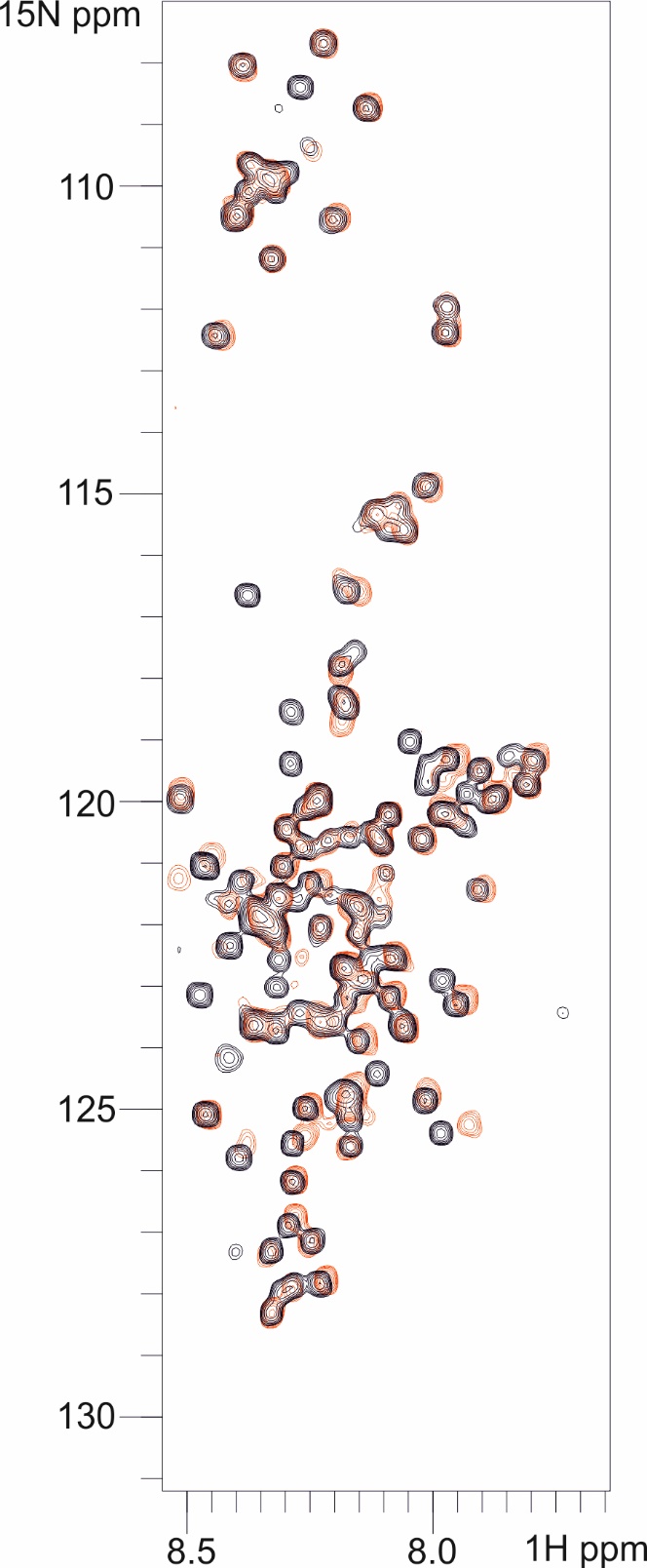
**

**Figure S6** ^1^H-^15^N HSQC spectrum of ^15^N- αSynA53T (100μM) (black) in the presence of unlabelled CypD (final concentration of 1mM) (red) in 20mM phosphate buffer, 20mM NaCl, pH 6.5, 298K. The addition of CypD induces selective chemical shift perturbations in the spectrum of ^15^N- αSynA53T, similar to the ones observed for wild-type αSyn.
